# Effects of dietary supplementation with prebiotics and *Pediococcus acidilactici* on gut health, transcriptome, microbiota, and metabolome in Atlantic salmon (*Salmo salar* L.) after seawater transfer

**DOI:** 10.1186/s42523-023-00228-w

**Published:** 2023-02-11

**Authors:** Anusha K. S. Dhanasiri, Alexander Jaramillo-Torres, Elvis M. Chikwati, Torunn Forberg, Åshild Krogdahl, Trond M. Kortner

**Affiliations:** 1 Department of Paraclinical Sciences, Faculty of Veterinary Medicine, Norwegian University of Life Sciences (NMBU), Ås, Norway; 2 Biomar RD, Trondheim, Norway

**Keywords:** Functional ingredients, Prebiotics, Probiotics, FOS, GOS, Gut microbiota, *Pediococcus acidilactici*, Metabolomics, Transcriptomics, Atlantic salmon

## Abstract

**Background:**

Given the importance of gut microbiota for health, growth and performance of the host, the aquaculture industry has taken measures to develop functional fish feeds aiming at modulating gut microbiota and inducing the anticipated beneficial effects. However, present understanding of the impact of such functional feeds on the fish is limited. The study reported herein was conducted to gain knowledge on performance and gut health characteristics in post-smolt Atlantic salmon fed diets varying in content of functional ingredients. Three experimental diets, a diet containing fructo-oligosaccharides (FOS), a diet with a combination of FOS and *Pediococcus acidilactici* (BC) and a diet containing galacto-oligosaccharides (GOS) and BC, were used in a 10-weeks feeding trial. A commercial diet without functional ingredients was also included as a control/reference. Samples of blood plasma, mucosa and digesta were subjected to microbiota, transcriptome and metabolome profiling for evaluation of the diet effects.

**Results:**

No significant growth differences were observed between fish fed the supplemented diets, but FOS–BC fed fish showed significantly faster growth than the control fed fish. The microbiota results showed that the BC was present in both the digesta, and the mucosa samples of fish fed the FOS–BC and GOS–BC diets. Digesta-associated microbiota was altered, while mucosa-associated microbiota was relatively unaffected by diet. Replacing FOS with GOS increased the level of metabolites linked to phospholipid, fatty acid, carnitine and sphingolipid metabolism. Variation in metabolite levels between the treatments closely correlated with genera mainly belonging to *Firmicutes* and *Actinobacteria* phyla. The transcriptome analyses indicated diet effects of exchanging FOS with GOS on immune functions, oxidative defense and stress responses. No significant diet effect was observed on intestinal inflammation in the pyloric caeca or in the distal intestine, or on lipid accumulation in the pyloric caeca.

**Conclusions:**

Dietary supplementation with BC induced moderate effects on the microbiota of the digesta, while the effects of replacing FOS with GOS were more marked and was observed also for nutrient metabolism. Our data indicates therefore that the quality of a prebiotic may be of great importance for the effects of a probiotic on gut microbiota, function, and health.

**Supplementary Information:**

The online version contains supplementary material available at 10.1186/s42523-023-00228-w.

## Background

To be able to grow sustainably, the salmon aquaculture industry has during the last 2 decades moved away from the traditional high fishmeal/fish oil diets, by gradually increasing the use of plant raw materials and alternative sources of lipid. Dietary incorporation of functional ingredients is also gaining attention to improve the robustness of the fish. Gut microbiota is important for performance and well-being of the fish. Therefore, efforts have been made to develop functional feeds aiming at modulating the gut microbiota to induce anticipated beneficial effects. Several previous studies have been conducted to evaluate the effect of feeds supplemented with probiotics, prebiotics or synbiotics, i.e. combinations of pre and pro-biotics, for farmed fish species including Atlantic salmon [1–5]. However, further efforts are still needed to better understand the combined effect of those functional ingredients on gut microbiota, gut function and health, and overall performance of the fish.

Dietary supplementation of probiotic bacteria can modulate gut microbiota and gut immune responses in beneficial ways and contribute to the synthesis of nutrients, ultimately improving disease resistance and growth performance of the fish [1]. The lactic acid bacteria, *Pediococcus acidilactici* MA 18/5M, is among the most widely studied probiotic bacteria for farmed fish species [6–10] and has been reported to enhance gut mucosal and peripheral immunity*.* Prebiotics may also exert beneficial host effects, via stimulation of the growth and/or the activity of the gut microbial population [3]. Several studies have indicated beneficial effects in fish of prebiotics such as fructo-oligosaccharide (FOS), galacto-oligosaccharide (GOS), mannan-oligosaccharide, beta glucans and inulin [3, 4, 11]. On the other hand, a recent large-scale study with salmon under commercial farming conditions showed little or no effects of dietary supplementation of a mixture of nucleotides, yeast cell walls and essential fatty acids [12], but indicated that these specific functional ingredients may represent an energetic cost for the fish.

Synbiotics, a mixture of probiotic and prebiotic agents, can have beneficial effects on the host by improving the survival and implantation of probiotic and/or the growth and activity of the indigenous beneficial bacteria in the gut [13]. Therefore, an optimal combination of probiotics and prebiotics in a single product could elicit a superior effect, compared to the activity of each component alone [14]. Studies of application of synbiotics in aquaculture species have increased over the past years including some studies on salmonids [2, 5, 15, 16]. Dietary application of *P. acidilactici* and GOS has shown effects such as increased immune responses and disease resistance, microbiota and metabolic alterations in rainbow trout (*Oncorhynchus mykiss*) [17–19], increased growth in juvenile rockfish (*Sebastes schlegeli*) [6] and some effects on mucosal and serum immune parameters in common carp (*Cyprinus carpio*) fingerlings [20]. A few studies have reported effects of dietary inclusion with *P. acidilactici* and FOS such as modulation of gut microbiota and immunity in Atlantic salmon [7] and increased growth performance of Caspian roach (*Rutilus frisii kutum*) fry [21].

**Fig. 1:**
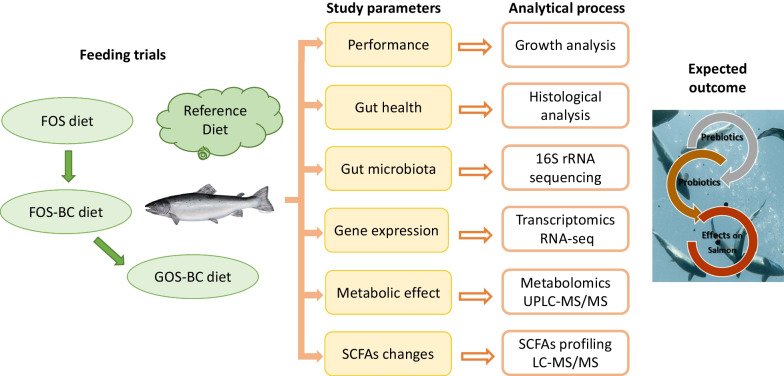
Schematic representation of experimental design. This study evaluated the effects of supplementation of *P. acidilactici* in diets for Atlantic salmon performance and gut health after transfer from freshwater to seawater. Fish were fed FOS alone (FOS diet) and FOS and GOS in combination with *P. acidilactici* (FOS–BC and GOS–BC diets respectively) and a commercial diet as a control/reference for 10 weeks. Six different parameters were analyzed using traditional and state-of-art-multi-omics techniques as detailed in the materials and methods section to investigate the effects of supplementing the diet containing the prebiotic FOS with the probiotic *P. acidilactici* (FOS–BSC vs. FOS) and replacing FOS in the FOS–BC diet with GOS (GOS–BC vs. FOS–BC) on post-smolt Atlantic salmon. Photograph. Geir Mogen, BioMar

Economically, Atlantic salmon is one of the most important farmed fish species worldwide [22]. The post-smolt stage (early marine phase) is one of the critical stages in Atlantic salmon life cycle [23]. Suppression of gut health [12] and alterations of gut microbiota [24] were reported in Atlantic salmon during early marine phase. In this stage functional feeds could play an important role in increasing survival, health, growth, and overall performance of the fish. Considering the importance of gut microbiota in modulating the gut health and ultimately overall health and performance of the fish, this study was conducted to evaluate effects in post-smolt Atlantic salmon of supplementing a diet containing the prebiotic FOS with the probiotic *P. acidilactici* (BC) and replacing FOS in the diet containing BC with GOS. A grower diet without functional ingredients was also included as a reference/control. An overview of the experimental set up and investigated endpoints is illustrated in Fig. 1 and detailed in the materials and methods section. A multi-omics analytical approach was chosen with microbiota, transcriptome and metabolome profiling. This study strengthens the knowledge basis of effects of use of functional feeds on fish by unveiling the complex interrelated associations among the gut microbiota–transcriptome–metabolites. The knowledge gain would also aid in optimizing the inclusion of functional diets into commercial feed formulations.

## Results

Detailed comparisons are made between the two pairs of treatment for which the cause of differences can be interpreted and discussed to achieve the goals of the study, i.e. fish fed the FOS and the FOS–BC diets and those fed the FOS–BC and GOS–BC diets. This approach will help us to understand the effects of supplementation of probiotic, BC, to a diet containing prebiotic, FOS, and the influence of alteration of prebiotic combined with BC.

### Performance data

**Fig. 2:**
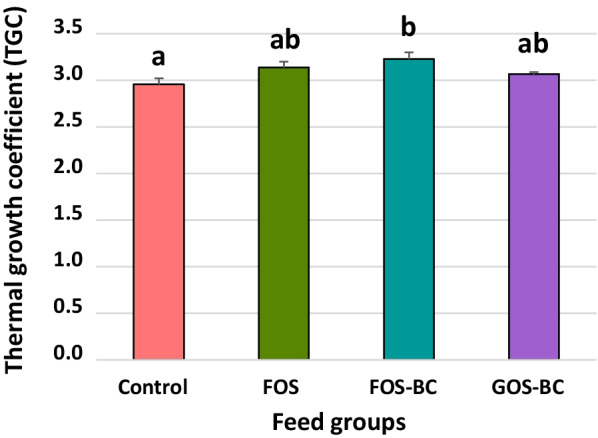
The thermal growth coefficient (TGC) of Atlantic salmon fed different diets. Post-smolt Atlantic salmon was fed a commercial diet as a control/reference and three experimental diets: FOS alone (FOS diet) and FOS and GOS in combination with *P. acidilactici* (FOS–BC and GOS–BC diets respectively) for 10 weeks. Values are mean of 210 fish per group. Error bars represent SEM (standard error of the mean). Different letters among values indicate statistically significant differences (q ≤ 0.05). Values sharing the same letters are not statistically significant. Significant difference observed only between the fish fed Control and FOS–BC diets (q ≤ 0.05)

The fish grew well throughout the experiment showing thermal growth coefficients (TGCs) averaging about 3.1 (Fig. 2). Fish in the FOS–BC group grew significantly faster than those in the control/reference group, showing TGCs of 3.23 and 2.96, respectively, during the 10 weeks of feeding. However, no significant differences in growth were observed with the supplementation of BC to FOS diet (FOS–BC vs. FOS) or after replacing FOS with GOS in FOS–BC diet (GOS–BC vs. FOS–BC). Feed intake and feed conversion ratios, which averaged 847 g ± 8 (SEM) and 1.12 ± 0.02 (SEM), respectively, showed no significant differences among the four treatments.

### Gut histology

**Fig. 3:**
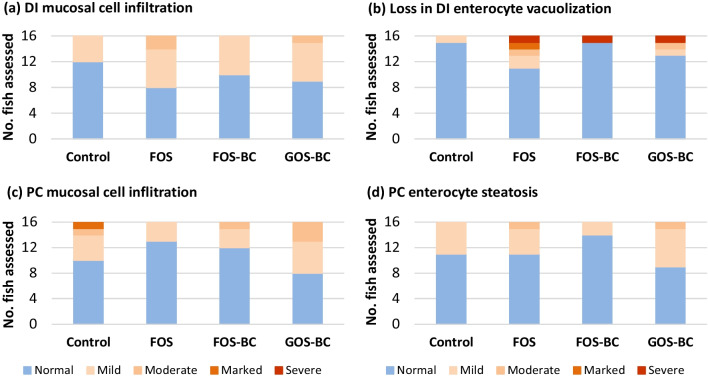
Histomorphological evaluation of distal intestine (DI) and pyloric caeca (PC) of Atlantic salmon. Number of fish scored as normal, mild moderate, marked, or severe for selected histomorphological of **a** distal intestine inflammatory cell infiltration (*p* = 0.638), **b** loss of distal intestine enterocyte vacuoles (*p* = 0.097), **c** inflammatory cell infiltration of the pyloric caeca mucosa (*p* = 0.529), and **d** lipid accumulation (steatosis) in pyloric caeca enterocytes (*p* = 0.437). *p* values represent outcomes of an ordinal logistic regression for differences in histology score outcomes between the treatment and the reference group, control

The distal intestine and pyloric caeca of the fish from the four treatments showed largely normal morphological characteristics, but some individuals from all diet groups showed abnormal morphology that ranged from mild to severe. Figure 3a and b illustrate the observations made regarding signs of inflammation in the distal intestine, i.e. regarding cell infiltration and loss of distal intestine enterocyte vacuoles, respectively. The results showed no significant differences between treatments. The same was observed regarding infiltration of inflammatory cells in mucosa and lipid accumulation (steatosis) in pyloric caeca, (i.e. inflammation and steatosis, Fig. 3c and d, respectively). The gut histological parameters were not affected by either supplementation of BC to FOS diet or after replacing FOS with GOS in the FOS–BC diet.

### Gut microbiota profiling

#### The absolute bacterial DNA levels

Bacterial DNA levels measured by qPCR analysis did not show significant differences between any of the three experimental diets. However, the variation between samples within treatment was large (Additional File 1: Fig. S1). Bacterial DNA levels in digesta were, in general, higher than the levels in mucosa.

#### Alpha diversity

Results regarding alpha diversity, i.e. number of different ASVs within a sample, measured as observed ASVs and Shannon indices, are presented in Additional File 1: Fig. S2a and b for digesta and S2c and S2d for mucosa. In the digesta samples, alpha diversity showed differing trend among the treatments (observed ASVs: *p* = 0.07 and Shannon: *p* = 0.08). However, pairwise comparisons indicated a significant difference between fish fed GOS–BC diet and FOS–BC diet (observed ASVs: *p* = 0.02 and Shannon: *p* = 0.005). The mucosa samples did not show significant diet effects among the fish fed different diets.

#### Beta diversity

**Fig. 4:**
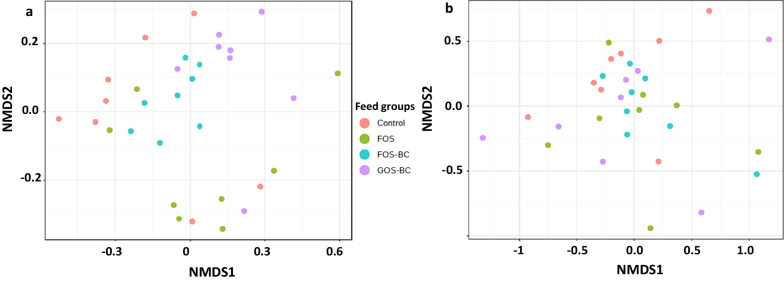
NMDS plots based on Bray–Curtis dissimilarity matrix showing beta diversity at ASV level. Beta diversity in the distal intestine digesta (**a**) and mucosa (**b**) of the Atlantic salmon fed with a control/reference diet and three experimental diets: FOS, FOS–BC and GOS–BC. The whole bacterial community of each sample is represented by a dot on the figure. Samples with similar bacterial compositions are closer to each other. PERMANOVA statistics for digesta: F value: 2.07; R-squared: 0.18; *p* value: 0.03; and [NMDS] Stress = 0.13. PERMANOVA statistics for mucosa: F value: 0.66; R-squared: 0.07; *p* value: 0.75; and [NMDS] Stress = 0.20. k value for NMDS analysis = 2

Beta diversity, i.e. differences in bacterial taxa between samples, taking into account taxa differences as well as the abundance of the taxa, was evaluated by PERMANOVA analysis based on Bray–Curtis dissimilarity matrix at ASV level. For the digesta samples (Fig. 4a), overall significant differences among treatments were observed (*p* = 0.03). The microbiota structure in fish from the FOS–BC treatment showed clear separation from those in the FOS treatment (*p* = 0.007). On the other hand, the microbiota in fish from the GOS–BC treatment clustered close to, but distinct from that of the FOS–BC treatment (*p* = 0.02). The mucosa samples (Fig. 4b) did not show significant differences in beta diversity among different treatments.

#### Taxonomic composition

**Fig. 5:**
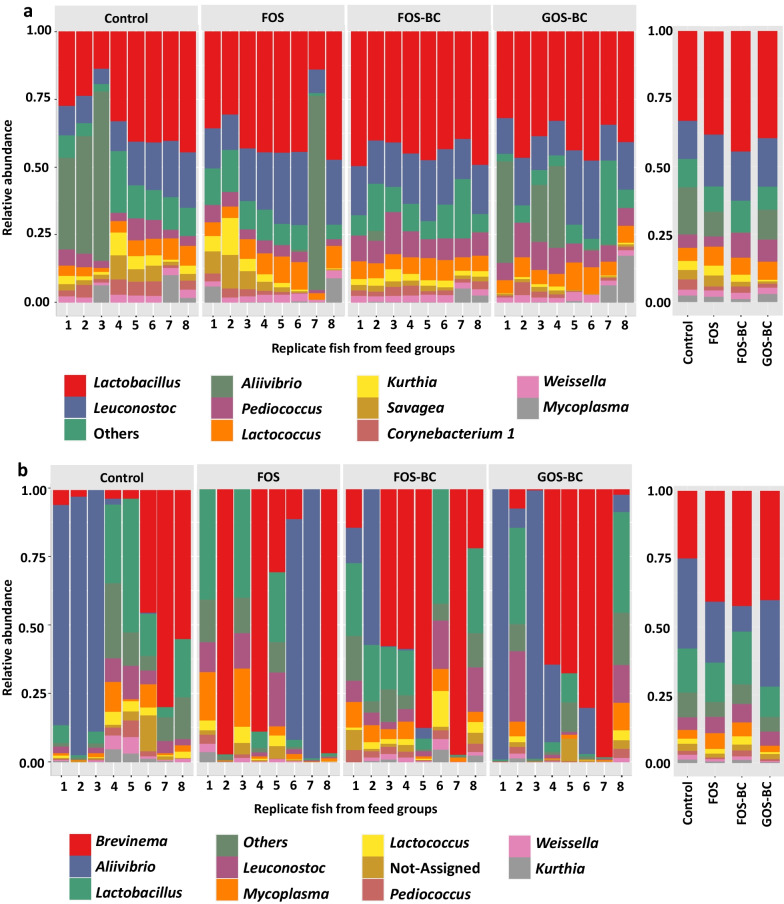
Top 10 most abundant genera of digesta (**a**) and mucosa (**b**) from distal intestine. The samples are grouped by feed groups: Atlantic salmon fed with a control/reference diet and three experimental diets: FOS, FOS–BC, and GOS–BC diets. The mean relative abundance of genera per feed group is presented on the right side

In the digesta, at the phylum level, *Firmicutes* dominate in most of the samples and *Firmicutes* and *Proteobacteria*, represented more than 90% of the average relative abundance in all treatments (Additional File 1: Fig. S3a). At the genus level, the lactic acid bacteria group, represented mainly by *Lactobacillus* and *Leuconostoc* comprised around 50% of the average relative abundance in all treatments (Fig. 5a). The complete list of genera in digesta which showed significant changes in their abundance among treatments are presented in Additional File 2: Tables S1. The number of differentially abundant genera in FOS–BC versus FOS comparison was 19, and 15 of them showed higher abundance in the FOS–BC fed fish. *Pediococcus* and *Staphylococcus* were among the genera showing increase. Fish fed the GOS–BC diet, compared to those fed the FOS–BC diet, showed reduction in 24 genera including *Kurthia*, *Savagea*, *Staphylococcus*, *Vagococcus* and *Peptostreptococcus*.

In the mucosa, the most abundant phyla were *Spirochaetes*, *Firmicutes* and *Proteobacteria.* Together they accounted for approximately 90% of averaged relative abundance in all the treatments (Additional File 1: Fig. S3b). The dominant genera in mucosa were *Brevinema*, *Aliivibrio* and *Lactobacillus* which comprised around 70% averaged relative abundance per feeding group (Fig. 5b).

**Fig. 6:**
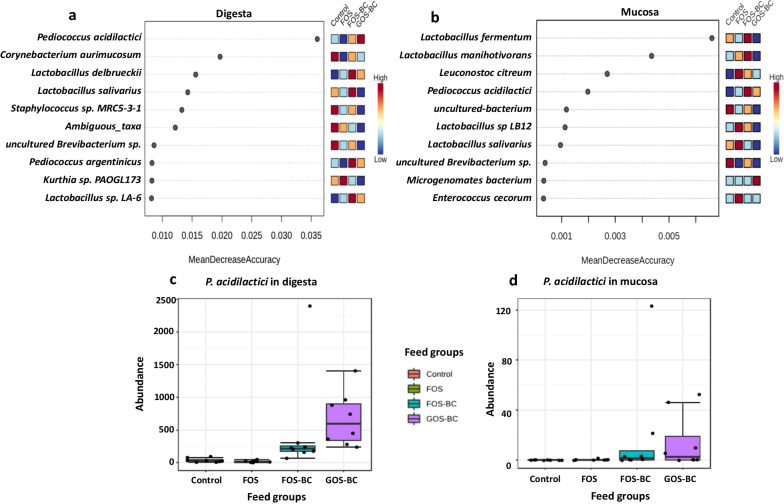
Random Forest importance plot indicating top 10 microbial species valuable for discriminating four treatments. Top 10 microbial species in digesta (**a**) and mucosa (**b**). The importance of the species is ordered from top to bottom and an estimate of their importance is indicated by the corresponding mean decrease accuracy. Color ranging from blue to red indicates the species abundance ranging from low to high i.e. blue color indicates low abundance and red color indicates high abundance. Box plots showing filtered absolute counts of *P. acidilactici* in digesta (**c**) and mucosa (**d**) which is important for separating fish in FOS–BC and GOS–BC from those in the control and FOS treatments. Note that the scale of y-axis is different for digesta (**c**) and mucosa (**d**) in box plots

We further employed the Random Forest model, a supervised machine-learning algorithm, for classification and identification of microbial taxa that differentiate among treatments. Random Forest model performed well for correctly predicting the microbial species of the replicates fish from four treatments in the digesta samples, but not in the mucosa samples, as indicated by 0.25 and 0.906 OOB (out of bag) error obtained, respectively (Additional File 2: Tables S2 and S3). Therefore, in the following, we mainly focus on digesta-associated microbiota. The model classified the treatments FOS–BC and GOS–BC quite precisely with 87.5% predicting accuracy for the digesta samples. In the digesta samples, the most important taxon which allowed discrimination of fish fed diets supplemented with BC from the other fish, was *P. acidilactici* (Fig. 6a). In the mucosa, it was the fourth most important discriminatory taxon (Fig. 6b). Both digesta (Fig. 6c) and mucosa (Fig. 6d), samples from the FOS–BC and GOS–BC diet fed fish had higher abundance of *P. acidilactici* compared to the FOS and control diet fed fish. Moreover, both digesta and mucosa samples from the fish fed the GOS–BC diet had higher abundance of *P. acidilactici* compared to the fish fed the FOS–BC diet (Fig. 6c, d). The abundance of *P. acidilactici* in digesta was substantially higher than its abundance in mucosa samples.

### Transcriptome profiling

The RNA-seq data showed raw read counts ranging from 20.4 to 42.8 million reads with an average count of 30.1 million per sample. Uniquely mapped reads ranged between 15 and 32 million among the samples having an 71% of average unique mapping efficiency.

#### Differently expressed genes (DEGs)

The global transcriptomic analysis revealed the highest number of DEGs (Benjamini–Hochberg adjusted *p* < 0.1, Table 1) in the GOS–BC treatment compared to the other treatments. Annotated DEGs among treatments are presented in Additional File 3. Transcriptomic changes in the distal intestine of fish fed the FOS–BC diet compared to those fed the FOS diet showed a low number of DEGs (27 up- and 6 down-regulated, Table 1, Additional File 3: File S1). Global transcriptome analysis showed major differences in the distal intestine between fish fed the GOS–BC diet and FOS–BC diet, 174 up- and 46 down-regulated in fish fed GOS–BC diet compared to those fed FOS–BC diet. Among the upregulated genes in fish fed with GOS–BC diet were cysteine knot cytokine members, interleukin 17 and receptors, *Il17a, il17a/f1* and *i17ra*; TNF superfamily members and receptors *tnfrsf1b*, *tnfrsf1*, *tnfrsf9a* and *tnfsf18*; beta trefoil cytokine family member *il-1rl*; and a number of chemokines (Additional File 3: File S2). The fish in the GOS–BC treatment also showed an increase in expression of transcripts of NADPH oxidases family of enzymes, dual oxidases (*duox* and *duox2*) and NADPH oxidase activator 1 (*noxo1a* and *noxo1b*) and key antioxidant enzyme, glutathione peroxidase 1b (*gpx1b*).

**Table 1:** Number of differentially expressed genes (DEGs) resulted from pairwise comparisons of treatments

Comparisons	Differentially expressed genes (DEGs) (q < 0.1, FC > 1.5)		
	Total	Upregulated	Downregulated
FOS–BC versus FOS	34	27	6
GOS–BC versus FOS–BC	220	174	46
FOS versus control	07	04	03
FOS–BC versus control	07	02	05
GOS–BC versus control	537	269	268

#### Gene ontology (GO) enrichment analysis

**Fig. 7:**
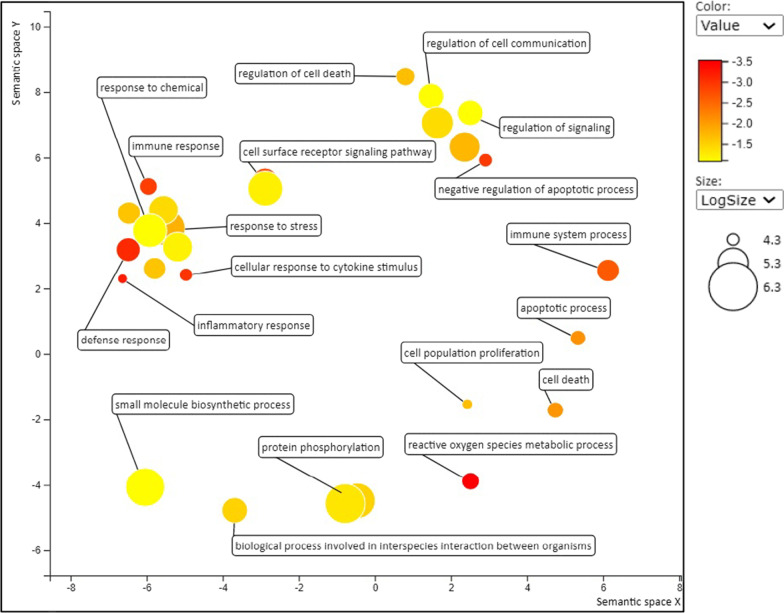
Non-redundant enriched gene ontology (GO) biological processes. Figure shows the enriched biological processes detected for the upregulated genes in Atlantic salmon fed the GOS–BC diet compared to fish fed the FOS–BC diet. Data are summarized as scatter plots using REVIGO tool. GO terms are marked with circles and plotted according to semantic similarities to other GO terms. The color of the circles ranging from yellow to red indicates the order of increase in log10 *p* value. Circle sizes are proportional to the respective frequencies of the GO terms (circles of more general terms are larger). Not all the terms are indicated in the figure due to the space limitations and the complete list of non-redundant enriched GO terms can be found in Additional File 2: Table S4

Results of GO enrichment analysis did not indicate enrichment of biological processes within the statistical criteria for the FOS–BS versus FOS comparison due to the low number of DEGs. Statistically enriched biological processes, as indicated by upregulation of genes, were observed only for GOS–BC versus FOS–BC and GOS–BC versus Control. The complete list of summarized GO terms generated from respective comparisons are available in Additional File 2: Table S4. The summarized GO terms generated from enriched nonredundant biological function GO terms are presented in Fig. 7 for upregulated genes in fish fed the GOS–BC diet compared to the FOS–BC diet. Among the enriched GO biological process terms were immune response, apoptotic process, inflammatory response, response to stress and reactive oxygen species metabolic process (Fig. 7).

### Metabolome profiling

The global metabolome profiling detected 747 and 655 metabolites in total, respectively in distal intestine digesta, and blood plasma samples collected from the various treatments. The number of significantly altered metabolites among fish fed different diets are presented in the Table 2.

**Table 2:** Number of significantly altered metabolites obtained from pairwise comparisons of treatments

Comparisons	Significantly altered metabolites in digesta (*p* ≤ 0.05)		Significantly altered metabolites in plasma (*p* ≤ 0.05)	
	Increased	Decreased	Increased	Decreased
FOS–BC versus FOS	14	13	03	19
GOS–BC versus FOS–BC	86	23	18	34
FOS versus Control	60	56	104	48
FOS–BC versus Control	63	63	65	60
GOS–BC versus Control	165	62	103	101

All the detected metabolites highlighting the significantly altered metabolites in each of the comparisons between treatments are presented in the Additional File 3: Files S4 and S5 for digesta and plasma, respectively. Although some differences were observed, many of the changes in plasma and digesta metabolites mirrored each other by dietary treatment (Additional File 3: Files S4 and S5). Among those were metabolites important for methylation of protein lysine and/or carnitine biosynthesis (such as N6-methyllysine, N6, N6, N6-trimethyllysine and deoxycarnitine) and microbiota-linked metabolism (*N*-methylhydantoin). Supplementation of BC to FOS resulted few significant effects (27 and 22 differentially abundant metabolites respectively in digesta and mucosa samples), generally scattered over the metabolic map, not showing clear effects on any metabolic pathway. On the other hand, replacement of FOS in the FOS–BC diet with GOS, significantly altered a high number of metabolites in both digesta and plasma (109 and 52 differentially abundant metabolites respectively in digesta and plasma samples). Unique for the GOS–BC treatment were high levels of long chain saturated, monounsaturated, and polyunsaturated fatty acids, as well as of branched fatty acids, most pronounced for digesta (Additional File 3: File S4). Among those metabolites were n−3 (eicosapentaenoic acid, EPA, 20:5n−3 and docosapentaenoic acid, DPA, 22:5n−3) and n−6 fatty acids (linoleic acid, 18:2n−6, eicosadienoic acid, 20:2n−6, arachidonic acid, 20:4n−6, adrenic acid, 22:4n−6 and dihomo-gamma-linolenic acid; 20:3n−6, DPA, 22:5n−6 and tetracosahexaenoic acid, 24:6n−3). The GOS–BC fed fish also showed increased levels in the digesta of acetylcarnitine, propionylcarnitine, butyrylcarnitine compared to FOS–BC fed fish, as well as compared to the other treatments. Levels of several sphingomyelins, ceramides and hexosylceramides were also increased distinctively in the GOS–BC fed fish compared to the FOS–BC fed fish and fish from the other treatments.

### Short chain fatty acid levels

The metabolome analyses of plasma samples did not show significant treatment effects, neither regarding the major SCFAs (acetic acid, butyric acid, and propionic acid) nor the minor (valeric acid and hexanoic acid, and branched short chain fatty acids, 2-methylbutyric acid, isobutyric acid and isovaleric acid) (Additional File 2: Table S5). On the other hand, in the digesta, butyric and valeric acid showed significantly lower values for the GOS–BC treatment compared to the control (Table 3). SCFAs in the digesta did not significantly change either with the addition of BC to FOS diet or replacement of FOS with the GOS in the FOS–BC diet.

**Table 3:** SCFA concentrations in distal intestinal digesta of the fish from four treatments

	SCFA concentrations in digesta of distal intestine (ng/ml)			
	Control	FOS	FOS–BC	GOS–BC
Acetic acid	8.4E+04 ± 3.9E+04	8.3E+04 ± 2.6E+04	6.5E+04 ± 1.8E+04	1.6E+05 ± 7. 5E+04
Butyric acid	83 ± 11^a^	68 ± 7^ab^	60 ± 4^ab^	54 ± 3^b^
Propionic acid	121 ± 17	101 ± 9	87 ± 7	88 ± 6
Valeric acid	41 ± 4^a^	34 ± 3^ab^	32 ± 7^ab^	28 ± 1^b^
Hexanoic acid	254 ± 22	225 ± 13	213 ± 1	203 ± 7
2-Methylbutyric acid	21 ± 4	16 ± 2	16 ± 2	12 ± 1
Isobutyric acid	25 ± 3	20 ± 1.5	21 ± 2	19 ± 1
Isovaleric acid	13 ± 2	14 ± 1	14 ± 1	12 ± 1

Mean value ± SEM are presented for n = 8 samples. Different letters among values indicate statistically significant differences (q ≤ 0.05). Values sharing the same letters are not statistically significant

### Associations between gut microbiota and metabolites

#### Correlation analysis

**Fig. 8:**
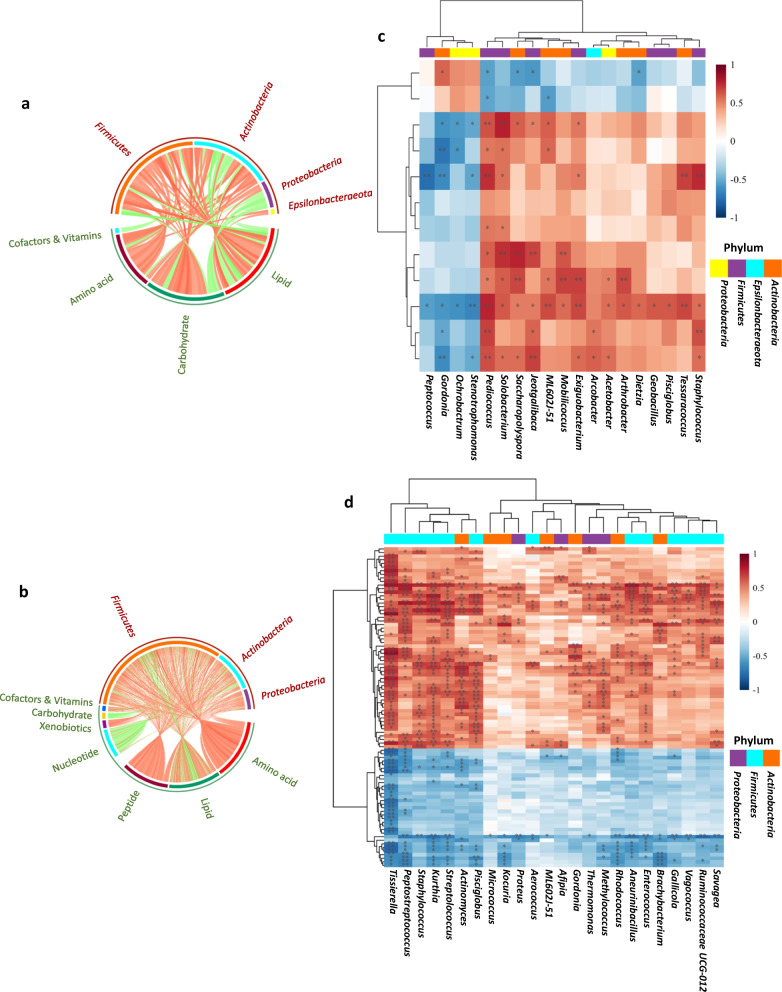
Circos plots (**a**, **b**) and heatmaps (**c**, **d**) showing associations analysis. Associations analysis performed between differentially abundant microbiota and metabolites in FOS–BC group compared to FOS group (**a**, **c**) and GOS–BC group compared to FOS–BC group (**b**, **d**) based on Spearman Correlation Analysis. Heatmaps show expansion of the results shown in the circos plots. Spearman's correlation, R, ranges between − 1 to 1. *p* < 0.05 indicates a statistically significant correlation. In circos plots, red and green lines specify positive and negative correlations, respectively. In heatmaps, red color and blue color indicate positive and negative correlations respectively. The darker color indicates the larger statistical significance. Symbol * and ** indicate *p* value for correlation coefficients smaller than 0.05 or 0.01, respectively. Correlations between differential microbiota and metabolites among the treatments including R and *p* values are presented in Additional File 4

The Spearman correlation analysis showed significant differences in specific microbe–metabolite correlations between the treatments. In the correlation analyses 436 digesta metabolites with the human metabolome database (HMDB) IDs were included. The circos plot and the heat map for microbe–metabolite correlations in digesta samples from comparisons between FOS–BC and FOS, and GOS–BC and FOS–BC treatments are presented in Fig. 8. Heatmaps show expansion of the results shown in the circos plots. In the heatmaps, statistically significant results (*p* < 0.05) are indicated with asterisks. Correlations values (R) and *p* values for the specific microbe–metabolite correlations are presented in Additional File 4.

Regarding the results of associations analysis of gut microbiota and metabolite for the comparison between FOS–BC and FOS treatments, circos plot showed that 4 different classes of metabolites, carbohydrates, cofactors and vitamins, amino acids, and lipids were closely correlated with genera belonging to *Firmicutes*, *Actinobacteria*, *Proteobacteria* and *Epsilonbacteoeota* phyla (Fig. 8a). As shown in the heatmap (Fig. 8c), the 15 genera which increased in the FOS–BC treatment compared to FOS treatment showed correlation with 12 significantly changed metabolites from the same comparison and found a number of significantly positive correlations (between 10 and 11). Genus *Pediococcus* showed positive and significant associations with 10 metabolites including lactose, ergosterol, chiro-inositol and ribose (Additional File 4: File S1).

The comparison between the GOS–BC and FOS–BC treatments showed the highest number of associations between microbiota and metabolites, and most of them were significant. Circos plot showed that seven different classes of metabolites including nucleotides, carbohydrates, peptides, cofactors and vitamins, xenobiotics, amino acids, and lipids were closely correlated with genera mainly belonging to *Firmicutes*, *Actinobacteria* and *Proteobacteria* phyla (Fig. 8b). As shown in the heatmap (Fig. 8d), all the 24 decreased genera in GOS–BC treatment compared to FOS–BC treatment displayed positive correlation with several metabolites (between 54 and 56 metabolites). On the other hand, those genera showed negative correlations with n−3 and n−6 polyunsaturated fatty acids (Additional File 4: File S2).

#### Supervised multivariate analysis

Supervised multivariate analysis on the combined data matrix of microbiota (at genus level) and metabolites in the digesta with the OPLS-DA method pointed out some separation between FOS–BC and FOS treatments as indicated by the first component (Additional File 1: Fig. S4a). On the other hand, it showed a clear separation between GOS–BC and FOS–BC treatments (Additional File 1: Fig. S4b).

Variable importance plot (not shown) based on the OPLS-DA model was used to identify differential microbes and metabolites contributing to the separation of one group compared to the other (Variable Importance on Projection, VIP values > 1 and correlation coefficients *p* < 0.05). The list of microbiota and metabolites fulfilling the above statistical criteria in each comparison between dietary treatments are presented in the Additional File 5. The FOS–BC treatment showed 41 separating factors (19 microbial genera and 22 metabolites), from the FOS treatment (Additional File 5: File S1). The genus *Pediococcus* was identified as an important variable in FOS–BC group distinguishing it from the FOS group. VIP plot identified 115 separating factors (20 microbial genera and 95 metabolites) in the GOS–BC group compared to the FOS–BC group (Additional File 5: File S2). Further, Genus *Pediococcus* was identified as an important variable in both FOS–BC and GOS–BC treatments from the control and the FOS treatment. Among the metabolites, lactose, ergosterol and deoxycarnitine found to be separating factors for FOS and FOS–BC groups, as well as GOS–BC and FOS–BC groups.

## Discussion

### Effects of supplementation with *P. acidilactici* to the FOS diet

Improved growth was observed for fish fed the FOS and *P. acidilactici* diet when compared to the commercial control diet. However, it is not possible for us to evaluate whether the synbiotic treatment was the causative factor for the observed improvements in growth, since the experimental diets also contained elevated levels of vitamin C and E, beta glucan and nucleotides, and had a partial substitution of standard fish meal with krill meal. A previous study reported no significant change in growth when Atlantic salmon were fed a diet supplemented with the same synbiotic combination [7]. As such, it is possible that the improved growth observed in the current study was caused by other dietary supplements besides the synbiotics, or by a combined effect.

The observation in the present study showing that alteration in diet composition, in this case supplementation with *P. acidilactici* to the FOS containing diet, modified the digesta-associated microbiota in the distal intestine of post-smolt Atlantic salmon is in line with other recent observations in salmon [25]. The same is the case for the results regarding the mucosa-associated microbiota, which showed resistance towards dietary changes, again confirming the results from Li et al. [25] and supported by the findings from Abid et al. [7]. Moreover, our findings that, irrespective of diet, genera *Lactobacillus* and *Leuconostoc* dominated in the digesta and *Brevinema*, *Aliivibrio* and *Lactobacillus* dominated in mucosa are also strengthening previous findings which indicate that these bacterial groups are among the core microbiota in digesta and mucosa in post-smolt Atlantic salmon [25–27]. Alpha diversity, or species richness of the digesta-associated microbiota in the fish fed FOS–BC diet did not differ significantly from those fed FOS diet indicating that supplementation of *P. acidilactici* to the FOS diet did not alter the number of ASVs in digesta. Similar to the present observation, a previous study [7] has reported that dietary application of FOS and *P. acidilactici* did not induce significant changes in alpha diversity in the digesta-associated microbiota in the distal intestine of Atlantic salmon. On the other hand, the fact that fish fed FOS–BC diet showed a significantly different beta diversity in the digesta from that of the fish fed FOS diet, indicates an ability of *P. acidilactici* when in combination with FOS to modulate the bacterial composition by altering abundance of the various bacteria. As expected, *P. acidilactici* showed relatively higher abundance in both the digesta and mucosa samples of fish fed FOS–BC diet compared to those fed FOS diet, suggesting strengthened establishment in the gut. Previous studies have demonstrated the ability of *P. acidilactici* to populate the distal intestine of Atlantic salmon when used as dietary supplementation [10] or in combination with FOS [7]. In the present study, *P. acidilactici* was found to be the key factor separating gut microbiota of fish fed FOS–BC diet from that of the fish fed FOS diet indicating its importance for modulating the gut microbiota profile. The *P. acidilactici* was also the key factor for distinguishing the digesta-associated microbiota profiles of FOS–BC fed fish from those in the fish fed control diet.

Since gut microbiota plays an important role in shaping the fecal metabolome, we expected that the combined evaluation of microbiota and metabolomics data could give us a better understanding of the possible functional implications of the diets used in the present study. We analyzed the global metabolite signature and SCFA levels in both digesta and blood plasma, expecting that microbiota and metabolic associations may give local as well as systemic effects [28, 29]. However, only a few metabolites showed significant difference between the fish fed FOS–BC and FOS diets, demonstrating that the supplementation of *P. acidilactici* to the FOS diet had a minor impact on the metabolome of both the gut content and the systemic circulation. As a consequence, associations between gut microbiota and metabolites were also few. The general lack of strong host responses after *P. acidilactici* supplementation to the FOS diet was also observed on the transcriptional level.

### Effects of replacement of GOS for FOS in the FOS–BC diet

No previous published studies have compared GOS–BC supplementation with FOS–BC supplementation to fish feeds. However, supplementation of GOS–BC to a control diet was previously reported to increase growth performance and lower FCR in rainbow trout fingerlings [30] and juvenile rockfish, *Sebastes schlegeli* [6], whereas a more recent study reported no significant changes in growth parameters in rainbow trout upon application of the same synbiotic combination in rainbow trout diets [19]. The results of the present study are in line with the results of the latter work.

#### Effects on microbiota

Replacing FOS with GOS induced a reduction in alpha diversity i.e. a decrease in the number of ASVs present, as well as a change in beta diversity indicating a shift of abundance of some of the bacteria. Fish in the GOS–BC treatment showed increased relative abundance of *P. acidilactici* compared to the FOS–BC treatment. This increase in both the digesta and mucosa indicates enhanced establishment of *P. acidilactici* when in combination with GOS relative to FOS. Increased abundance of *P. acidilactici* was also reported in Rainbow trout treated with GOS in combination with the same probiotic species [19]. *Pediococcus acidilactici* strains are known to produce bacteriocins, pediocins, which may exert antagonistic effects towards a variety of bacteria including both gram negative and positive species [31, 32]. Therefore, decreased alpha diversity and the reduced abundance of several genera observed when replacing FOS with GOS in the diet could potentially be a result of increased antagonistic effects exerted by *P. acidilactici* when combined with GOS. However, this should be further investigated with measurements of pediocins in the digesta, mucosa and blood samples as reduced relative abundance of other genera could also simply be due to the increased relative abundance of *P. acidilactici*.

#### Impact on the metabolome of digesta and blood plasma

Replacing FOS with GOS increased levels of short, medium, and long chain acyl-carnitines in both digesta and plasma. This suggests that GOS could directly influence or act as a substrate for the gut microbiota to supply the intestinal mucosa and the body with compounds having important functions in lipid transport and metabolism. Carnitine and its acyl esters (acyl-carnitines) are essential for transport of fatty acids across the outer and inner mitochondrial membranes, for the mitochondrial beta-oxidation of long-chain fatty acids, as well as for maintenance of the ratio of acetyl-CoA/CoA [33, 34]. On the other hand, gut bacteria can utilize carnitine for protection against osmotic stress [35].

The increase in several sphingolipids, including sphingomyelin and interrelated products such as ceramide, and hexosylceramides in the fish fed GOS–BC diet suggests that GOS may affect and possibly improve various barrier functions. Sphingolipids, mainly ceramide, act as signaling molecules and are involved in diverse processes including epithelial integrity, cell growth and death, apoptosis, immunity, and inflammation [36, 37]. As they are important in orchestration of immune responses (cytokine release, inflammatory responses and initiation of apoptosis of the infected cell) and eliminating invading pathogens [37], many pathogens have developed strategies to exploit host cell sphingolipid pathways to change the sphingolipid balance to facilitate their colonization [38]. Therefore, it is possible that the increase in sphingolipid levels in GOS–BC fed fish might trigger an immune response. The transcriptome results seem to indicate such an effect as explained below.

Most of the SCFAs levels were quite similar in the fish of the four treatments. The exceptions were butyric acid and valeric acid which showed a reduction in fish fed GOS–BC diet compared to those fed control. SCFAs are among the most important microbial metabolites in the gut and are reported to exert multiple beneficial effects on vertebrates by involvement in energy homeostasis and healthy immune responses [39, 40]. However, only a few studies investigating pre- pro- or synbiotic applications in fish have reported effects on SCFA production, and the observations are quite different from those in the present study. For example dietary application of *Enterococcus faecalis* in Javanese carp, *Puntius gonionotus* increased the intestinal propionic and butyric acid, but not acetic acid [41]; and *Alcaligenes* sp. increased intestinal acetic acid, but not butyric acid levels in Malaysian Mahseer, *Tor tambroides* [42]*.* Mammalian studies have indicated that formation of SCFAs by intestinal bacteria is regulated through many different host, environmental, dietary and microbiological factors with substrate availability, microbial species composition and intestinal transit time playing a larger role [43]. Therefore, relatively similar SCFA concentrations observed among the fish in the three treatments and control possibly indicate that SCFA regulation is quite stable in the Atlantic salmon even if the dietary and microbial compositions differed among the treatments. However, this needs to be further investigated.

#### Impact on the transcriptome

The observed transcriptomic changes upon the FOS to GOS exchange, i.e. upregulation of genes coding for a number of cytokines and/or their receptors (*Il17a*, *il17a/f1, i17ra*, *tnfrsf1b*, *tnfrsf1*, *tnfrsf9a*, *tnfsf18* and *il-1rl*) indicate alterations in communication between innate and adaptive immune systems [44, 45]. The increase in expression of the toll-like receptor 18 gene (*tlr18*), important for bacterial pathogen recognition [46], and of the antibacterial peptide gene (*hepc1*) may indicate effects of the exchange of prebiotic on immune functions important for disease resistance. The same regards the increase in expression of transcripts (*duox*, *duox2*, *noxo1a* and *noxo1b*) important for reactive oxygen species generation and innate host defense pathways on mucosal surfaces, cellular signaling, regulation of gene expression and cell differentiation [47, 48]. The GOS–BC treatment also displayed increased expression of the key antioxidant enzyme, *gpx1b*, involved in protection of the fish from oxidative stress.

Upregulation of genes involved in immune and other defense mechanisms does not necessarily mean increased resistance towards infection diseases or other stressors—it could also representant an adaptation to the diet without important implications for disease resistance. Before conclusions regarding effects on robustness of the fish can be made, follow-up studies involving infectious challenge or other stress challenge studies should be conducted. The effects on immune and other defense genes can also possibly be due to an increase in production of pediocin with antimicrobial properties by the highly abundant *P. acidilactici* when in combination with GOS. On the other hand, activation of defense mechanisms may also be a sign of inflammatory responses. However, the histological appearance of the distal intestine did not indicate altered state of inflammation which was evaluated as mild to moderate for all treatments. The mechanism underlying the alteration in the transcriptome may be the combined effects of (a) the direct influence of GOS, and (b) the indirect influence caused by the action of microbiota on the GOS and (c) effects of altered metabolite production in the microbiota linked to the alteration in beta diversity. Support for the suggestion of beneficial effect of GOS on disease resistance is found in studies with rainbow trout in which a combination of GOS and *P. acidilactici* increased antioxidant defense biomarkers, innate immune responses, and resistance to streptococcosis [17, 18].

#### Correlations between impacts on microbiota, metabolome and transcriptome

Replacing FOS with GOS in the FOS–BC diet showed significant impacts on gut microbiota and metabolite associations. Spearman correlation analysis revealed that metabolites including nucleotides, carbohydrates, peptides, cofactors and vitamins, xenobiotics, amino acids, and lipids were closely correlated with genera mainly belonging to *Firmicutes*, *Actinobacteria* and *Proteobacteria* phyla. Previous studies in fish and mammals have reported the involvement of gut microbiota in lipid metabolism and energy homeostasis [49, 50] and de novo synthesis of essential amino acids and vitamins [51, 52]. This suggests that supplementation of GOS and *P. acidilactici* in the diet could have modulated gut microbiota associated with some of those functions in the post-smolt Atlantic salmon in the present study as well. Further, increased transcripts and metabolite levels related to immunomodulatory effects could also potentially link to the increased abundance of *P. acidilactici* when in combination with GOS.

## Conclusions

This study reports effects on growth performance, gut health, microbiota, transcriptome, metabolome, and their associations in post-smolt Atlantic salmon fed diets containing the prebiotic FOS, a combination of FOS and the probiotic *P. acidilactici*, or a combination of GOS and *P. acidilactici*. No significant effects of these dietary alterations were detected on growth or histomorphological appearance of the gut. Supplementation with *P. acidilactici* to the FOS containing diet altered digesta associated microbiota to some degree, whereas the mucosa-associated microbiota seemed relatively resistant to such dietary modulation. This probiotic also induced moderate effects in some of the assessed components of the metabolome and transcriptome. Replacing FOS with GOS in FOS–BC diet induced several, clear effects on many of the observed biomarkers which may indicate that GOS induces important effects on the microbiota, metabolome in the digesta as well as the endogenous metabolism, as well as on the mucosal metabolism and function. However, those alterations did not significantly impact the growth performance of GOS–BC group. Further infection challenge and stress studies are needed to ascertain the efficacy of dietary application of GOS and *P. acidilactici* along with functional ingredient mixes as an immune stimulant strategy against disease outbreaks and stressful events.

## Materials and methods

Experimental design, study parameters and analytical procedures used to evaluate the effect of functional seawater transfer diets for Atlantic salmon are illustrated in the Fig. 1 and explained in the subsequent sections.

### Feeding trial

A sea water feeding trial was conducted with post-smolt Atlantic salmon at LetSea research facility in Dønna, Norway from 29/05/2018 to 16/09/2018, following the Norwegian laws regulating the experimentation with live animals.

Atlantic salmon with average weight 172 ± SEM 0.89 g were randomly assigned to 16 net pens (5 × 5 × 5 m) with 300 fish each. Four feeds were prepared by Biomar AS, a control diet based on standard grower feed recipes and three experimental diets. The experimental diets contained elevated vitamin C and E, beta glucan and nucleotides, and had a partial substitution of standard fish meal with krill meal. The experimental diets were further supplemented with either; prebiotic fructo-oligosaccharide (FOS, 0.1%), FOS (0.1%) and Bactocell (0.03%) (FOS–BC); or galacto-oligosaccharide (1.0%) and Bactocell (0.03%) (GOS–BC) (Table 4). Bactocell (Lallemand Inc., Cardiff, UK) is authorized by the European Union for the use in fish and shrimp [53] and has already been used in salmon fry and freshwater stage diets. All feeds were produced at Biomar Feed technology Center in Brande, Denmark. Four randomly distributed pens were allocated for each dietary group. Fish were fed above mentioned four feeds: acclimatization diets (3, 5 mm pellets) during the first 5 weeks following seawater transfer, and then the trial diets (5 mm pellets) for 10 additional weeks. Bulk weights for each pen were registered at the end of the acclimatization period and the 10-week feeding trial period to determine start and end weight of the experimental fish. During the experimental period, average seawater temperature of 12.4 ± 1.8 °C, salinity of 31.9 ± 0.7 ppt and oxygen of 10.0 ± 1.1 mg/l were reported.

**Table 4:** Composition of experimental diets for post-smolt Atlantic salmon

Diet composition (g/100 g)	Trial feeds (5 mm pellet size)			
	Control	FOS	FOS–BC	GOS–BC
Fish meal	15.0	15.0	15.0	15.0
Soya SPC	11.0	11.0	11.0	11.0
Wheat Gluten	7.2	8.0	8.0	8.0
Maize gluten	5.0	5.0	5.0	5.0
Pea protein	15.0	15.0	15.0	15.0
Guar meal	8.0	7.0	7.0	7.0
Wheat	11.0	10.8	10.9	10.0
Fish oil	13.2	11.5	11.5	11.5
Rapeseed oil	10.4	11.1	11.1	11.1
Vit + min + AA	4.3	4.9	4.9	4.9
Yttrium	0.1	0.1	0.1	0.1
FOS	–	0.1	0.1	–
GOS	–	–	–	1.0
Bactocell			0.03	0.03
Water change	− 0.1	0.5	0.5	0.5
Analyzed moisture (%)	5.8	5.4	5.7	6
Energy (bomb calorimetry, MJ/kg)	24.2	24.2	23.8	24.1
Crude FAT (%)	28.5	27.9	27.6	28.1
Crude protein (%)	43.2	43.5	43.9	43

Beta glucan, nucleotides and krill were added only to the experimental diets in equal amounts

At the end of the feeding trial, four fish were randomly taken from each net pen, anesthetized with tricaine methanesulfonate (MS222®; Argent Chemical Laboratories, Redmond, WA, USA), weighed individually and euthanized by a sharp blow to the head. Blood samples were drawn from the caudal vein using heparinized syringes and placed on ice before plasma collection. Plasma was collected after centrifugation at 2000*g* for 10 min (4 °C) and snap frozen in liquid N2. After cleaning the exterior of each fish with 70% ethanol, the distal intestine was aseptically removed, opened longitudinally and digesta was collected into a 50 ml sterile centrifuge tube. The digesta was mixed thoroughly with a spatula and aliquots were transferred into 1.5 ml sterile Eppendorf tubes and snap frozen in liquid N_2_ and stored at − 80 °C for the analysis of the digesta-associated intestinal microbiota and metabolomic profiling. The mid-section of the same distal intestine was excised and rinsed 3 times in sterile phosphate-buffered saline. Subsequently, the tissue was transversely divided into 3 pieces, respectively, for histological evaluation (fixed in 4% phosphate-buffered formaldehyde solution for 24 h and transferred to 70% ethanol for storage), RNA-Sequencing (preserved in RNAlater solution and stored at − 20 °C) and mucosa-associated intestinal microbiota analysis (snap frozen in liquid N_2_ and stored at − 80 °C).

The performance of the fish in each dietary group was calculated using the thermal growth coefficient and specific growth rate, which are considered as good predictors of salmon growth [54]. Statistical analysis of growth parameters among the treatments was performed by one-way ANOVA after checking the fulfillment of all the pertinent assumptions, normality of the distribution and homogeneity of variances. Pairwise comparisons were analyzed using Tukey's honestly significant different (HSD) test, and q ≤ 0.05 was considered as statistically significant.

### Histological analysis

The gut tissue sections (total of 64 fish, n = 16 per dietary group, n = 4 fish randomly selected from each of the 4 pens allocated for a dietary group) of pyloric caeca and distal intestine were evaluated by light microscopy with focus on the characteristic morphological changes of soybean meal-induced enteritis (SBMIE) in Atlantic salmon distal intestine, that consist of shortening of mucosal fold length, increase in width and inflammatory cell infiltration of the submucosa and lamina propria, and reduction in enterocyte supranuclear vacuolization. Additionally, for the pyloric caeca, changes in the vacuolization of the intestinal epithelial cells were evaluated. Normally, little to no vacuolization is present in the intestinal epithelial cells of the pyloric caeca and mid intestine. Increased vacuolization (or hyper-vacuolization) is observed in fish affected by the so-called lipid malabsorption syndrome (LMS) that manifests in its advanced form as ‘floating feces’ (steatorrhea).

The degree of change was graded using a scoring system with a scale of 0–4 where 0 represented normal; 1, mild; 2, moderate; 3, marked, and 4, severe changes. The histological evaluation was conducted randomly and blind, and assignment of individual samples to the treatments was obtained after the evaluation was completed.

Differences in histological scores for the evaluated morphological characteristics of the intestinal tissue were analyzed for statistical significance using ordinal logistic regression run in the R statistical package (version 3.6.3; 2020) within the RStudio interphase (version 1.3.1093; 2020). Differences were examined based on odds ratios of the different treatments having different histology scores compared to the reference diet. Control was used as the reference.

### Microbiota analysis

#### DNA extraction

For analysis of the distal intestinal microbiota, a total of 32 fish samples were used. Two fish were randomly selected from each of the 4 pens allocated for a dieatary group to have n = 8 fish per diatary group. The DNA was extracted from respective digesta and mucosa samples following the protocol of QIAamp Fast DNA Stool Kit (Qiagen, Crawley, UK) with some modification as suggested by Knudsen et al. [55]. Samples were pre-processed with a bead-beating protocol of three times in the Fastprep at 6.5 m/s for 30 s with a mix of beads (120 mg acid-washed glass beads (150–212 μm) and 240 mg Zirconium oxide beads (1.4 mm). For quality control of the microbiota profiling protocol, along with the each of the DNA extraction batch, two ‘blanks’ (without any sampling materials) and two ‘positive controls’ i.e. mock (microbial community standard from Zymo-BIOMICS™, Zymo Research, California, USA) were included. The mock contains 8 bacteria (*Pseudomonas aeruginosa*, *Escherichia coli*, *Salmonella enterica*, *Lactobacillus fermentum*, *Enterococcus faecalis*, *Staphylococcus aureus*, *Listeria monocytogenes*, *Bacillus subtilis*) and 2 yeasts (*Saccharomyces cerevisiae*, *Cryptococcus neoformans*).

#### PCR amplification of V1–V2 region of the 16S rRNA gene

PCR amplification was carried out using 27F (5′ AGAGTTTGATCMTGGCTCAG 3′), and 338R-I (5′ GCWGCC TCCCGTAGGAGT 3′) and 338R-II (5′ GCWGCCACCCGTAGGTGT 3′) to have about 300 bp amplicons [26]. PCRs were carried out in 25 μl reactions with 12.5 μl of Phusion® HighFidelity PCR Master Mix (Thermo Scientific, CA, USA); 1 μM of forward and reverse primers, and 1 μl template DNA. Undiluted and 1:2 diluted templates were used, respectively, from the digesta and mucosa. The PCR conditions were as follows: initial denaturation at 98 °C for 7 min followed by initial 10 cycles with denaturation at 98 °C for 30 s, annealing temperature decreasing from 63 to 53 °C for 30 s at each temperature and extension at 72 for 30 s; followed by 25 further cycles with denaturation at 98 °C for 30 s, annealing at 53 °C for 30 s, and extension at 72 °C for 30 s; followed by a final extension at 72 °C for 10 min. Negative PCR controls were included by replacing the template DNA with molecular grade water. PCR was performed in duplicate, pooled, and examined by 1.5% agarose gel electrophoresis.

#### Library preparation and sequencing

Library preparation of the products from amplicon PCR was performed using the Quick-16S™ NGS Library Prep Kit (Zymo Research) following the instructions from the producer. Briefly, PCR products were first enzymatically cleaned up followed by a PCR to add barcodes. Subsequently, the libraries were quantified by qPCR, pooled, and purified. A representative number of individual libraries were evaluated for DNA quality in Agilent Bioanalyzer 2100 system (Agilent Technologies, California, USA). The final pooled library was then denatured and diluted to 8 pM and sequenced on Illumina MiSeq platform with Miseq Reagent Kit v3 (600-cycle) (Illumina) to generate paired-end read. 20% of 8 pM PhiX control was added as an internal control.

#### Bacterial DNA quantification by qPCR

As an extra measure to identify contaminating sequences, qPCR was performed to quantity 16S rRNA gene in the diluted DNA templates (samples, blanks, and mocks) used for the amplicon PCR. The qPCR assays were performed using a universal primer set (forward, 5′-CCA TGA AGT CGG AAT CGC TAG-3′; reverse, 5′-GCT TGA CGGGCG GTG T-3′) as described previously [56, 57]. The qPCR was performed using the LightCycler 96 (Roche Applied Science, Basel, Switzerland) in a 10 µl reaction volume; 2 µl of PCR-grade water, 1 µl diluted DNA template, 5 µl LightCycler 480 SYBR Green I Master Mix (Roche Applied Science) and 1 µl (3 µM) of each primer. The qPCR program used as follows; an initial enzyme activation step at 95 °C for 2 min, 45 three-step cycles of 95 °C for 10 s, 60 °C for 30 s and 72 °C for 15 s, and a melting curve analysis at the end. Quantification cycle (Cq) values were determined using the second derivative method [58] and bacterial DNA standards were used as inter-plate calibrators and the inter-plate calibration factor was calculated as described previously [59].

#### Bioinformatics analysis of microbiota sequencing data

This was performed using QIIME2 version 2 [60, 61]. The demultiplexed paired-ended reads were denoised, trimmed and quality filtered using the DADA2 algorithm [62] in QIIME2. Primer sequences were trimmed off (forward reads, first 20bps; reverse reads, first 18bps) and the reads were truncated at the position where the median Phred quality crashed (forward reads, at position 290 bp; reverse reads, at position 238 bp) and low-quality reads were filtered out. Chimeric sequences were removed after merging the reads. The taxonomy was assigned to resulting amplicon sequence variants (ASVs) tables by a Scikitlearn Naive Bayes machine-learning classifier [63], which was trained on the SILVA 132 99% ASVs [64] that were trimmed to exclusively include the regions of 16S rRNA gene amplified by the primers used in the current study. Filtering of ASVs table was performed using q2-feature-table plugin in Qiime2. ASVs assigned as chloroplast and mitochondria were removed from ASVs table. The ASVs table was then filtered to remove ASVs that were without a phylum-level taxonomic assignment or appeared in only one biological sample. Low abundance ASVs with total abundance of less than 2 across all the samples were also filtered out. Contaminant sequences were detected using control samples (negative PCR reactions, DNA extraction blanks and mocks) and bacterial DNA quantification data obtained from qPCR mentioned in the previous section, as suggested by Davies et al. [65]. In general, contaminants are frequently found in negative controls and blanks and show a negative correlation with the bacterial DNA concentration. Moreover, contaminants also can be foreign ASVs in mocks those are not belonging to the original included bacteria. In total 17 and 11 ASVs were removed from mucosa and digesta samples respectively based on their presence in mocks, extraction blanks and negative PCR controls, and their negative correlation with bacterial DNA concentration. The ASVs removed from mucosa samples belonged to the genera *Rhodoluna* (1 ASV), *Cutibacterium* (1 ASV), *Flavobacterium* (6 ASVs), *Afipia* (1 ASV), *Curvibacter* (2 ASVs), *Limnohabitans* (1 ASV), *Polynucleobacter* (1 ASV), *Ralstonia* (2 ASVs), *Undibacterium* (1 ASV) and *Pseudomonas* (1 ASV). On the other hand, the removed contaminants from digesta samples belonged to the genera *Flavobacterium* (6 ASVs), *Curvibacter* (2 ASVs), *Rhodoluna* (1 ASV), *Polynucleobacter* (1 ASV) and *Ralstonia* (1 ASV). After filtering, a total number of 1 075 and 385 ASVs were obtained for digesta and mucosa samples, respectively. The ASVs filtered from the raw ASVs table were also removed from the representative sequences. The final ASVs tables with taxonomy are presented in Additional File 6.

Diversity analysis was performed using q2-diversity plugin in Qiime2. To compute alpha and beta diversity indices, the ASVs tables were rarified at 28,295 and 15,655 reads for digesta and mucosa samples respectively in order to have an even number of reads across all the samples. The rarefaction curves based on observed ASVs for the digesta and mucosa samples from 32 fish and from each feed group are presented in Additional File 1: Figs. S5 and S6 for digesta and mucosa, respectively. Alpha diversity was calculated using observed species and Shannon`s diversity indices at ASVs level. Beta diversity was evaluated using Bray–Curtis at ASVs level followed by PERMANOVA analysis along with pairwise comparisons. MicrobiomeAnalyst package [66, 67] was used to analyze abundant taxa among treatments, Random Forest analysis, NMDS analysis and graphical presentations of data using ASVs tables.

### Global transcriptomic profiling

#### RNA sequencing

Total RNA was extracted from distal intestinal digesta of 28 fish (n = 7 per dietary group) from the 32 fish used for microbiota analysis using Invitrogen PureLink RNA Mini Kit with column based purification (Thermo Fisher Scientific, Waltham, USA), following the manufacturer’s protocol. Tissues were homogenized twice at 5000×*g* for 15 s with zirconium oxide beads (1.4 mm) using FastPrep-24™ (MP Biomedicals, Thermo Fisher Scientific, Waltham, USA). RNA integrity was checked using an Agilent 2200 TapeStation (Agilent Technologies, Santa Clara, USA), and RNA quantity and RNA purity were measured using Epoch Microplate Spectrophotometer (BioTeK Instruments, Winooski, USA).

Library preparation and RNA sequencing was performed by Norwegian National Sequencing Center (Oslo, Norway). Libraries were prepared using TruSeq® Stranded mRNA Library Prep kit with TruSeq RNA unique dual indexes in accordance with the manufacturer’s protocol (Illumina, San Diego, USA). Sequencing was performed on the Illumina SP Novaseq flow cell to yield 100 bp single end reads.

#### Bioinformatics analysis of RNA-seq data

After demultiplexing, raw sequencing data was processed for quality and adapter trimming using Cutadapt [68] with − q 25, 20, quality-base = 33, trim-n -m 20 parameters, followed by a further quality check with FastQC (https://www.bioinformatics.babraham.ac.uk/projects/fastqc/). Quality trimmed reads were mapped to the indexed Atlantic salmon genome, ICSASG v2 with refseq genes using HISAT2 package [69] in Norwegian e-Infrastructure for Life Sciences (NeLS) galaxy platform developed by ELIXIR Norway [70]. HTSeq [71] was used to compute gene expression values. Differentially expressed genes among the treatments were determined using DESeq2 [72] using the default parameters. DESeq2 performs differential expression analysis based on the negative binomial (Gamma-Poisson) distribution. The analysis is executed through 3 main steps; estimation of size factors, estimation of dispersion, and negative binomial generalized linear model fitting and Wald statistics [72]. DESeq2 uses un-normalized count data as input, and it internally corrects for library size. DESeq2 performs independent filtering by removing genes with low counts which are not likely to produce significant differences due to high dispersion. It uses the mean of normalized counts irrespective of the biological conditions for independent filtering [72]. By default, DESeq2 replaces outliers if the Cook’s distance is large for a sample. Differential expression was calculated for pairwise comparisons using un-transformed data. The differences were considered statistically significant when the adjusted *p* value (q) with the Benjamini–Hochberg procedure ≤ 0.1. For the visualization of DEGs in heatmaps, log transformed count data was used.

#### Functional annotation and gene ontology analysis of DEGs

Functional annotation of the DEGs was performed using g:Profiler online tool [73, 74] and manually inspecting the Ensembl (http://www.ensembl.org) and NCBI (https://www.ncbi.nlm.nih.gov/) data bases. Gene ontology enrichment analysis (GO) was carried out also with g:Profiler online tool. For the calculation of statistically significant enrichment, all the known genes of the Atlantic salmon in the Ensembl database (Ensembl 100, Ensemble genome 47) were considered and the threshold to determine GO terms was set as Benjamini–Hochberg FDR (False Discovery Rate) value of 0.1. Enriched GO terms were then summarized by removing redundant GO terms and visualized in semantic similarity-based scatterplots using REVIGO online tool [75].

### Short chain fatty acids and metabolites analysis

Targeted short chain fatty acids analysis and global untargeted metabolite profiling were performed by Metabolon, Inc. (Morrisville, USA). Plasma and digesta collected from the same 32 fish (n = 8 per dietary group) used for microbiota and transcriptomics analysis.

#### SCFA analysis

For the SCFA analysis, samples were spiked with stable labelled internal standards, homogenized, and subjected to protein precipitation. An aliquot of the supernatant was derivatized, then diluted and injected onto liquid chromatography-tandem mass spectrometry, LC–MS/MS system (Agilent 1290 LC system, Agilent Technologies Inc, Santa Clara, USA with AB Sciex QTrap 5500 system, AB Sciex, Framingham, USA). The mass spectrometer was operated in negative mode using electrospray ionization (ESI). The peak area of the individual analyte product ions was measured against the peak area of the product ions of the corresponding internal standards. Quantification was performed using a weighted linear least squares regression analysis generated from fortified calibration standards prepared immediately prior to each run. LC–MS/MS raw data were collected and processed using AB SCIEX software Analyst 1.6.2. Analyte concentrations that fell below and above the limit of quantitation were removed from the downstream analysis. From all the SCFAs analyzed, only 4 out of the 32 samples were below the quantitation for one SCFA, isobutyric acid.

#### Global metabolite profiling

Samples were prepared by automated Microlab STAR (Hamilton company, Reno, USA) system [76]. Metabolon inc. used ultraperformance liquid chromatography-tandem mass spectroscopy, UPLC-MS/MS (UPLC from Waters ACQUITY, Milford, USA and Q-Exactive mass spectrometer from Thermo Scientific, Waltham, USA), for the metabolite analysis. After protein precipitation, the resulting extract was aliquoted, and two aliquots were analyzed by separate reverse phase (RP)/UPLC-MS/MS methods with positive mode using ESI; one aliquot with RP/UPLC-MS/MS with negative mode using ESI; and one aliquot by hydrophilic interaction chromatography (HILIC)/UPLC-MS/MS with negative mode using ESI. Several controls were analyzed in concert with the experimental samples including a pooled matrix sample (and/or a pool of well-characterized human plasma) served as a technical replicate throughout the data set; extracted water samples served as process blanks; and a cocktail of QC standards (carefully selected not to interfere with the spiked endogenous compound into all the samples) to monitor instrument performance and aid in chromatographic alignment. Instrument variability and overall process variability were determined respectively by the standards and spiked endogenous compounds.

Raw data was extracted, peak-identified and QC processed using hardware and software developed by Metabolon [76, 77]. Metabolites were identified by automated comparison of the ion features in the experimental samples to a reference library of chemical standard entries that included retention time, molecular weight (*m/z*), preferred adducts, and in-source fragments as well as associated MS spectra and were quality controlled and curated to identify true chemical entities [76, 77]. Peaks were quantified using area-under-the-curve. Data normalization step was performed to correct variation resulting from instrument inter-day tuning differences.

#### SCFA and metabolite data analysis

Statistical analysis of changes in SCFA concentrations among the treatments were carried out using one-way ANOVA followed by Tukey HSD test after checking for the fulfillment of all pertinent assumptions for ANOVA. Changes in SCFAs considered statistically significant when q ≤ 0.05. For the metabolites data, originally normalized data (normalized to correct the variation due to instrument inter-day tuning differences) was rescaled to set the median equal to 1. Then missing values were imputed with the minimum. Welch’s *t*-test which allows for unequal variances was used to analyze changes in metabolite concentrations among the treatments and metabolite concentrations considered statistically significant when *p* ≤ 0.05.

### Correlation analysis of microbiota and metabolites

Correlation analysis of microbiota and metabolites was performed using M2IA online tool [78]. As per the requirement of the tool, only the metabolites with HMDB IDs (436 and 293 respectively for digesta and plasma), and ASVs table with taxonomic annotations and corresponding reference sequence file generated from QIIME2 analysis were used. Data was processed by filtering out both the microbiota and metabolic features with missing values found in more than 80% of samples and the relative standard deviation values less than 30%. Minimum value was selected to impute missing value for both data sets. For data normalization, the relative percentage of features calculated based on the total sum scaling was used. For the pair-wise comparisons of the treatments, Wilcoxon rank-sum test was used and the *p* < 0.05 was considered as statistically significant.

Spearman correlation analysis method was selected to analyze correlations between differentially abundant microbiota (genus level) and metabolite concentrations in one dietary group compared to the other. Spearman correlation analysis method was recommended by the developers of M2IA online tool as it outperforms other correlation analysis methods due to its overall performance regarding specificity, sensitivity, similarity, accuracy, and stability with different sparsity [79]. The coefficient values (R) ranged between − 1 and 1 and *p* < 0.05 was considered statistically significant. The results were visualized on circos plots and heatmaps to identify bacterial genera that were closely related with different classes of metabolites.

Supervised multivariate analysis first integrates two data matrix and then identifies differential variables which significantly contribute to the discrimination between two treatments. We selected the orthogonal partial least squares discriminant analysis (OPLS-DA) method in M2IA to identify the microbiota and metabolites having a significant role in discriminating one dietary group from the other. Variables of importance for group separation were identified and clarified with variable importance plot. Variables with VIP > 1 and correlation coefficient (corr.coeffs) *p* < 0.05 were considered statistically significant.

## Supplementary Information

**Additional file 1. Figure S1.** The absolute bacterial DNA levels quantified by qPCR. DNA levels in digesta samples (**a**) and mucosa samples (**b**) from each of the treatments. n = 8 fish per group. Error bars represent SEM. No significant differences (*p* ≤ 0.05) found among the treatments. **Figure S2.** The alpha diversity indices for digesta and mucosa at ASV level. Observed ASVs (**a**) and Shannon indices (**b**) for digesta and observed ASVs (**c**) and Shannon indices (**d**) for mucosa. p values obtained from Kruskal–Wallis analysis among the feed groups are presented above each graph. Each box plot contains 25% and 75% quartiles of the data set respectively at the lower and upper ends of the box. The vertical line inside the box indicates the median, and the ends of the whiskers indicate minimum and maximum values of the data. Black rectangle indicates mean value of the data and dots display values from individual fish. **Figure S3.** Top 10 most abundant phyla of digesta (**a**) and mucosa (**b**) from distal intestine. The samples are grouped by feed groups: Atlantic salmon fed with a control/reference diet and three experimental diets: FOS, FOS–BC, and GOS–BC diets. The mean relative abundance of phyla per feed group is presented on the right side. **Figure S4.** Orthogonal partial least squares discriminant analysis (OPLS-DA) score plots. OPLS-DA score plots of the combined data matrix of metabolome and microbiota in each of the FOS–BC (**a**) and GOS–BC (**b**) groups compared to FOS and FOS–BC groups, respectively. Each dot indicates an individual sample. **Figure S5.** The rarefaction curves based on observed ASVs for the digesta samples. Rarefaction curves for the digesta samples from 32 fish (**a**) and each feed group (**b**). Each Feed group contains 8 samples. The ASVs table was rarified at 28 295, which is the minimum number of reads detected in the digesta samples. **Figure S6.** The rarefaction curves based on observed ASVs for the mucosa samples. Rarefaction curves for the mucosa samples from 32 fish (**a**) and from each feed group (**b**). Each Feed group contains 8 samples. The ASVs table was rarified at 15 655 reads, which is the minimum number of reads detected in the mucosa samples.

**Additional file 2: Table S1.** Significantly changed bacterial genera resulted from pairwise comparisons of treatments. **Table S2.** Random Forest confusion matrix for digesta-associated microbiota. **Table S3.** Random Forest confusion matrix for mucosa-associated microbiota. **Table S4.** Summarized enriched biological process GO terms produced using REVIGO tool for DEGs in GOS–BC group. **Table S5.** SCFA concentrations in blood plasma from four treatments.

**Additional file 3: File S1.** List of differentially expressed annotated genes in FOS–BC group compared to the FOS group. **File S2.** List of differentially expressed annotated genes in GOS–BC group compared to the FOS–BC group. **File S3.** List of differentially expressed annotated genes in GOS–BC group compared to the control group. **File S4.** Detected metabolites in digesta highlighting differential abundance in pairwise comparisons between treatments. **File S5.** Detected metabolites in plasma highlighting differential abundance in pairwise comparisons between treatments.

**Additional file 4: File S1.** The specific microbe–metabolite correlations in FOS–BC group compared to FOS group. **File S2.** The specific microbe–metabolite correlations in GOS–BC group compared to FOS–BC group. **File S3.** The specific microbe–metabolite correlations in FOS group compared to the control group. **File S4.** The specific microbe–metabolite correlations in FOS–BC group compared to the control group. **File S5.** The specific microbe–metabolite correlations in GOS–BC group compared to the control group.

**Additional file 5: File S1.** Variables of importance identified by V-plot to discriminate FOS–BC group from the FOS group. **File S2.** Variables of importance identified by V-plot to discriminate GOS–BC group from the FOS–BC group. **File S3.** Variables of importance identified by V-plot to discriminate FOS group from the control group. **File S4.** Variables of importance identified by V-plot to discriminate FOS–BC group from the control group. **File S5.** Variables of importance identified by V-plot to discriminate GOS–BC group from the control group.

**Additional file 6: File S1.** ASVs table for digesta samples. **File S2.** ASVs table for mucosa samples.

## Data Availability

16S rRNA sequencing and RNA-seq data are publicly available at the NCBI Sequence Read Archive (SRA) with the accession numbers SUB8676898 and SUB8572237 respectively, under the Bioproject PRJNA679207.
