# Supplementary material for: Effects of dietary supplementation with prebiotics and Pediococcus acidilactici on gut health, transcriptome, microbiota, and metabolome in Atlantic salmon (Salmo salar L.) after seawater transfer
Source: Anim Microbiome. 2023 Feb 11;5:10. doi: 10.1186/s42523-023-00228-w (PMC9921345; doi:10.1186/s42523-023-00228-w)
Supplement: Supplementary file 1 — Additional file 1. Figure S1. The absolute bacterial DNA levels quantified by qPCR. DNA levels in digesta samples (a) and mucosa samples (b) from each of the treatments. n = 8 fish per group. Error bars represent SEM. No significant differences (p ≤ 0.05) found among the treatments. Figure S2. The alpha diversity indices for digesta and mucosa at ASV level. Observed ASVs (a) and Shannon indices (b) for digesta and observed ASVs (c) and Shannon indices (d) for mucosa. p values obtained from Kruskal–Wallis analysis among the feed groups are presented above each graph. Each box plot contains 25% and 75% quartiles of the data set respectively at the lower and upper ends of the box. The vertical line inside the box indicates the median, and the ends of the whiskers indicate minimum and maximum values of the data. Black rectangle indicates mean value of the data and dots display values from individual fish. Figure S3. Top 10 most abundant phyla of digesta (a) and mucosa (b) from distal intestine. The samples are grouped by feed groups: Atlantic salmon fed with a control/reference diet and three experimental diets: FOS, FOS–BC, and GOS–BC diets. The mean relative abundance of phyla per feed group is presented on the right side. Figure S4. Orthogonal partial least squares discriminant analysis (OPLS-DA) score plots. OPLS-DA score plots of the combined data matrix of metabolome and microbiota in each of the FOS–BC (a) and GOS–BC (b) groups compared to FOS and FOS–BC groups, respectively. Each dot indicates an individual sample. Figure S5. The rarefaction curves based on observed ASVs for the digesta samples. Rarefaction curves for the digesta samples from 32 fish (a) and each feed group (b). Each Feed group contains 8 samples. The ASVs table was rarified at 28 295, which is the minimum number of reads detected in the digesta samples. Figure S6. The rarefaction curves based on observed ASVs for the mucosa samples. Rarefaction curves for the mucosa samples from 32 fish (a) and [file 42523_2023_228_MOESM1_ESM.docx]

Additional File 1

**Figure S1. The absolute bacterial DNA levels quantified by qPCR**. DNA levels in digesta samples (a) and mucosa samples (b) from each of the treatments. n=8 fish per group. Error bars represent SEM. No significant differences (p ≤ 0.05) found among the treatments.

**Figure S2. The alpha diversity indices for digesta and mucosa** **at ASV level.** Observed ASVs (a) and Shannon indices (b) for digesta and observed ASVs (c) and Shannon indices (d) for mucosa. p values obtained from Kruskal-Wallis analysis among the feed groups are presented above each graph. Each box plot contains 25% and 75% quartiles of the data set respectively at the lower and upper ends of the box. The vertical line inside the box indicates the median, and the ends of the whiskers indicate minimum and maximum values of the data. Black rectangle indicates mean value of the data and dots display values from individual fish.

**Figure S3.** **Top 10 most abundant phyla of digesta (a) and mucosa (b) from distal intestine.** The samples are grouped by feed groups: Atlantic salmon fed with a control/reference diet and three experimental diets: FOS, FOS-BC, and GOS-BC diets. The mean relative abundance of phyla per feed group is presented on the right side.

**Figure S4. Orthogonal partial least squares discriminant analysis (OPLS-DA) score plots**. OPLS-DA score plots of the combined data matrix of metabolome and microbiota in each of the FOS-BC (a) and GOS-BC (b) groups compared to FOS and FOS-BC groups, respectively. Each dot indicates an individual sample.

**Figure S5. The rarefaction curves based on observed ASVs for the digesta samples.** Rarefaction curves for the digesta samples from 32 fish (a) and each feed group (b). Each Feed group contains 8 samples. The ASVs table was rarified at 28 295, which is the minimum number of reads detected in the digesta samples.

**Figure S6. The rarefaction curves based on observed ASVs for the mucosa samples.** Rarefaction curves for the mucosa samples from 32 fish (a) and each feed group (b). Each Feed group contains 8 samples. The ASVs table was rarified at 15 655 reads, which is the minimum number of reads detected in the mucosa samples.
