# Supplementary material for: Effects of dietary supplementation with prebiotics and Pediococcus acidilactici on gut health, transcriptome, microbiota, and metabolome in Atlantic salmon (Salmo salar L.) after seawater transfer
Source: Anim Microbiome. 2023 Feb 11;5:10. doi: 10.1186/s42523-023-00228-w (PMC9921345; doi:10.1186/s42523-023-00228-w)
Supplement: Supplementary file 2 — Additional file 2: Table S1. Significantly changed bacterial genera resulted from pairwise comparisons of treatments. Table S2. Random Forest confusion matrix for digesta-associated microbiota. Table S3. Random Forest confusion matrix for mucosa-associated microbiota. Table S4. Summarized enriched biological process GO terms produced using REVIGO tool for DEGs in GOS–BC group. Table S5. SCFA concentrations in blood plasma from four treatments. [file 42523_2023_228_MOESM2_ESM.docx]

**Table S1.** Significantly changed bacterial genera resulted from pairwise comparisons of treatments

| **FOS-BC *vs* FOS** | **GOS-BC *vs* FOS-BC** |  |
| --- | --- | --- |
|  |  |  |
| **Increased** | **Decreased** |  |
| *Pediococcus* | *Savagea* |  |
| *Staphylococcus* | *Kurthia* |  |
| *Pisciglobus* | *Staphylococcus* |  |
| *Geobacillus* | *Vagococcus* |  |
| *Tessaracoccus* | *Peptostreptococcus* |  |
| *Mobilicoccus* | *Enterococcus* |  |
| *Exiguobacterium* | *Streptococcus* |  |
| *Solobacterium* | *Gallicola* |  |
| *ML602J-51* | *Aneurinibacillus* |  |
| *Dietzia* | *Methylococcus* |  |
| *Arthrobacter* | *Kocuria* |  |
| *Jeotgalibaca* | *Brachybacterium* |  |
| *Acetobacter* | *Micrococcus* |  |
| *Arcobacter* | *Proteus* |  |
| *Saccharopolyspora* | *Tissierella* |  |
|  | *Ruminococcaceae UCG-012* |  |
| **Decreased** | *Rhodococcus* |  |
| *Gordonia* | *ML602J-51* |  |
| *Ochrobactrum* | *Aerococcus* |  |
| *Stenotrophomonas* | *Pisciglobus* |  |
| *Peptococcus* | *Actinomyces* |  |
|  | *Thermomonas* |  |
|  | *Afipia* |  |
|  | *Gordonia* |  |
|  |  |  |
|  |  |  |
| **FOS *vs* Control** | **FOS-BC *vs* Control** | **GOS-BC *vs* Control** |
|  |  |  |
| **Increased** | **Increased** | **Increased** |
| *Ochrobactrum* | *Lactobacillus* | *Pediococcus* |
| *Stenotrophomonas* | *Pediococcus* | *W5053* |
| *Gordonia* | *Propionibacterium* | *Paraclostridium* |
|  | *ML602J-51* |  |
| **Decreased** | *Paraclostridium* | **Decreased** |
| *Glutamicibacter* | *Psychrilyobacter* | *Corynebacterium 1* |
| *Brevibacterium* | *Saccharopolyspora* | *Savagea* |
| *Aerococcus* | *Mycobacterium* | *Kurthia* |
| *Exiguobacterium* |  | *Vagococcus* |
| *Mobilicoccus* | **Decreased** | *Staphylococcus* |
| *Jeotgalicoccus* | *Glutamicibacter* | *Kocuria* |
| *Pseudogracilibacillus* | *Brevibacterium* | *Enterococcus* |
| *Carnobacterium* | *Globicatella* | *Peptostreptococcus* |
| *Arcobacter* | *Jeotgalicoccus* | *Aneurinibacillus* |
| *Jeotgalibaca* | *Pseudogracilibacillus* | *Methylococcus* |
|  | *Carnobacterium* | *Glutamicibacter* |
|  |  | *Brevibacterium* |
|  |  | *Aerococcus* |
|  |  | *Globicatella* |
|  |  | *Brachybacterium* |
|  |  | *Proteus* |
|  |  | *Ruminococcaceae UCG-012* |
|  |  | *Microbacterium* |
|  |  | *Jeotgalicoccus* |
|  |  | *Sanguibacter* |
|  |  | *Pseudogracilibacillus* |
|  |  | *Carnobacterium* |
|  |  | *Hathewaya* |
|  |  |  |

Statistically significant changes in bacterial genera (p < 0.05) in distal intestine digesta samples were obtained by the pair-wise comparisons of the feed groups, using Wilcoxon rank-sum test with M2IA online tool.

**Table S2.** Random Forest confusion matrix for digesta-associated microbiota

|  | Control | FOS | FOS-BC | GOS-BC | Class error |
| --- | --- | --- | --- | --- | --- |
| Control | **5** | 1 | 0 | 2 | 0.375 |
| FOS | 2 | **5** | 0 | 1 | 0.375 |
| FOS-BC | 0 | 0 | **7** | 1 | 0.125 |
| GOS-BC | 0 | 0 | 1 | **7** | 0.125 |

The OOB (out of bag) error is 0.25

**Table S3.** Random Forest confusion matrix for mucosa-associated microbiota

|  | Control | FOS | FOS-BC | GOS-BC | Class error |
| --- | --- | --- | --- | --- | --- |
| Control | **1** | 0 | 4 | 3 | 0.875 |
| FOS | 1 | **0** | 3 | 4 | 1.0 |
| FOS-BC | 3 | 2 | **1** | 2 | 0.875 |
| GOS-BC | 0 | 5 | 2 | **1** | 0.875 |

The OOB (out of bag) error is 0.906

**Table S4.** Summarized enriched biological process GO terms produced using REVIGO tool for DEGs in GOS-BC group

| **Term_ID** | **Description** | **Adjusted p-value (q)*** |
| --- | --- | --- |
|  |  |  |
| **Upregulated genes- GOS-BC group compared to FOS-BC group** | | |
| GO:0006915 | apoptotic process | 7.04E-03 |
| GO:0044419 | biological process involved in interspecies interaction between organisms | 2.94E-02 |
| GO:0008219 | cell death | 7.88E-03 |
| GO:0008283 | cell population proliferation | 1.90E-02 |
| GO:0007166 | cell surface receptor signaling pathway | 1.69E-03 |
| GO:0070887 | cellular response to chemical stimulus | 5.90E-02 |
| GO:0071345 | cellular response to cytokine stimulus | 9.57E-04 |
| GO:0006952 | defense response | 7.69E-04 |
| GO:0006955 | immune response | 1.19E-03 |
| GO:0002376 | immune system process | 2.14E-03 |
| GO:0006954 | inflammatory response | 5.77E-04 |
| GO:0035556 | intracellular signal transduction | 5.64E-02 |
| GO:0043066 | negative regulation of apoptotic process | 1.10E-03 |
| GO:0048519 | negative regulation of biological process | 3.71E-02 |
| GO:0048523 | negative regulation of cellular process | 1.66E-02 |
| GO:0016310 | phosphorylation | 4.52E-02 |
| GO:0012501 | programmed cell death | 7.04E-03 |
| GO:0006468 | protein phosphorylation | 2.94E-02 |
| GO:0072593 | reactive oxygen species metabolic process | 3.14E-04 |
| GO:0010646 | regulation of cell communication | 7.56E-02 |
| GO:0010941 | regulation of cell death | 1.96E-02 |
| GO:0023051 | regulation of signaling | 7.84E-02 |
| GO:0009607 | response to biotic stimulus | 2.19E-02 |
| GO:0042221 | response to chemical | 7.58E-02 |
| GO:0009605 | response to external stimulus | 3.53E-02 |
| GO:0051707 | response to other organisms | 2.19E-02 |
| GO:0006950 | response to stress | 1.46E-02 |
| GO:0044283 | small molecule biosynthetic process | 7.56E-02 |
|  |  |  |
| **Upregulated genes- GOS-BC group compared to control group** | |  |
| GO:0044281 | small molecule metabolic process | 1.84E-04 |
| GO:0072593 | reactive oxygen species metabolic process | 2.82E-03 |
| GO:0006082 | organic acid metabolic process | 3.82E-03 |
| GO:0006631 | fatty acid metabolic process | 7.31E-03 |
| GO:0006955 | immune response | 7.31E-03 |
| GO:0009605 | response to external stimulus | 7.31E-03 |
| GO:0044419 | biological process involved in interspecies interaction between organisms | 1.21E-02 |
| GO:0040011 | locomotion | 1.21E-02 |
| GO:0007166 | cell surface receptor signaling pathway | 1.45E-02 |
| GO:0048870 | cell motility | 1.58E-02 |
| GO:0051674 | localization of cell | 1.58E-02 |
| GO:0032787 | monocarboxylic acid metabolic process | 1.58E-02 |
| GO:0006508 | proteolysis | 1.58E-02 |
| GO:0006950 | response to stress | 1.58E-02 |
| GO:1901135 | carbohydrate derivative metabolic process | 1.84E-02 |
| GO:0019318 | hexose metabolic process | 1.99E-02 |
| GO:0005996 | monosaccharide metabolic process | 2.34E-02 |
| GO:0006915 | apoptotic process | 2.44E-02 |
| GO:0044262 | cellular carbohydrate metabolic process | 2.57E-02 |
| GO:0008219 | cell death | 2.65E-02 |
| GO:0009607 | response to biotic stimulus | 2.65E-02 |
| GO:0051707 | response to other organisms | 2.65E-02 |
| GO:0006952 | defense response | 2.87E-02 |
| GO:0042981 | regulation of apoptotic process | 3.06E-02 |
| GO:0071345 | cellular response to cytokine stimulus | 3.07E-02 |
| GO:0002376 | immune system process | 3.14E-02 |
| GO:0006928 | movement of cell or subcellular component | 3.14E-02 |
| GO:0010941 | regulation of cell death | 3.14E-02 |
| GO:0022402 | cell cycle process | 3.52E-02 |
| GO:0019725 | cellular homeostasis | 3.77E-02 |
| GO:0006486 | protein glycosylation | 4.00E-02 |
| GO:0070085 | glycosylation | 4.37E-02 |
| GO:0006629 | lipid metabolic process | 4.39E-02 |
| GO:1903047 | mitotic cell cycle process | 4.39E-02 |
| GO:0009100 | glycoprotein metabolic process | 4.40E-02 |
| GO:0046394 | carboxylic acid biosynthetic process | 4.92E-02 |
| GO:0006897 | endocytosis | 6.08E-02 |
| GO:0070887 | cellular response to chemical stimulus | 6.40E-02 |
| GO:1901137 | carbohydrate derivative biosynthetic process | 6.98E-02 |
| GO:0042221 | response to chemical | 9.91E-02 |
|  |  |  |

* Adjusted p-value obtained from g:Profiler functional annotation tool

**Table S5.** SCFA concentrations in blood plasma of the fish from four treatments

|  | SCFA concentrations in blood plasma (ng/ml) | | | |
| --- | --- | --- | --- | --- |
|  | Control | FOS | FOS-BC | GOS-BC |
| Acetic acid | 187 ± 51 | 127 ± 31 | 240 ± 57 | 196 ± 27 |
| Butyric acid | 30 ± 1.5 | 29 ± 4 | 29 ± 2 | 27 ± 1 |
| Propionic acid | 72 ± 20 | 46 ± 5 | 64 ± 15 | 60 ± 9 |
| Valeric acid | 15.6 ± 0.6 | 15 ± 0.7 | 15.3 ± 0.4 | 15 ± 1 |
| Hexanoic acid | 35 ± 2 | 34 ± 1.8 | 33 ± 2 | 36 ± 3 |
| 2-Methylbutyric acid | 53 ± 1 | 52 ± 2 | 51 ± 2 | 51 ± 2 |
| Isobutyric acid | 6.5 ± 0.7 | 11 ± 2 | 9 ± 2 | 11.3 ± 0.6 |
| Isovaleric acid | 3.2 ± 0.6 | 5 ± 1 | 4 ±0.5 | 4.8 ± 0.5 |

Mean value ± SEM are presented for n = 8 samples

There were no significant changes observed among the treatments
